# Total Arterial Coronary Bypass Graft Surgery is Associated with Better Long-Term Survival in Patients with Multivessel Coronary Artery Disease: a Systematic Review with Meta-Analysis

**DOI:** 10.21470/1678-9741-2020-0653

**Published:** 2021

**Authors:** Sérgio C. Rayol, Jef Van den Eynde, Luiz Rafael P. Cavalcanti, Antonio Carlos Escorel Neto, Arian Arjomandi Rad, Andrea Amabile, Wilson Botelho Filho, Arjang Ruhparwar, Konstantin Zhigalov, Alexander Weymann, Dario Celestino Sobral Filho, Michel Pompeu B. O. Sá

**Affiliations:** 1 Division of Cardiovascular Surgery, Pronto-Socorro Cardiológico de Pernambuco - PROCAPE, Recife, Pernambuco, Brazil.; 2 University of Pernambuco - UPE, Recife, Pernambuco, Brazil.; 3 Department of Cardiovascular Diseases, Research Unit of Cardiac Surgery, University Hospitals Leuven, Leuven, Belgium.; 4 Imperial College London School of Medicine, London, England.; 5 Department of Cardiac Surgery, University of Chicago Medicine, Chicago, United States of America.; 6 Instituto do Coração - InCor, Universidade de São Paulo - USP, São Paulo, São Paulo, Brazil.; 7 Department of Thoracic and Cardiovascular Surgery, West German Heart and Vascular Center Essen, University Hospital of Essen, University Duisburg-Essen, Essen, Germany.

**Keywords:** Coronary Artery Bypass, Publication Bias, Data Management, Meta-Analysis, Propensity Score, Confidence Intervals

## Abstract

**Introduction:**

The benefit of total arterial revascularization (TAR) in coronary artery bypass grafting (CABG) remains a controversial issue. This study sought to evaluate whether there is any difference on the long-term results of TAR and non-TAR CABG patients.

**Methods:**

The Medical Literature Analysis and Retrieval System Online (MEDLINE), Excerpta Medica dataBASE (EMBASE), Cochrane Central Register of Controlled Trials (CENTRAL/CCTR), Clinical Trials.gov, Scientific Electronic Library Online (SciELO), Literatura Latino-Americana e do Caribe em Ciências da Saúde (LILACS), and Google Scholar databases were searched for studies published by October 2020. Randomized clinical trials and observational studies with propensity score matching comparing TAR *versus* non-TAR CABG were included. Random-effects meta-analysis was performed. The current barriers to implementation of TAR in clinical practice and measures that can be used to optimize outcomes were reviewed.

**Results:**

Fourteen publications (from 2012 to 2020) involving a total of 22,746 patients (TAR: 8,941 patients; non-TAR: 13,805 patients) were included. The pooled hazard ratio (HR) for long-term mortality (over 10 years) was lower in the TAR group than in the non-TAR group (random effect model: HR 0.676, 95% confidence interval 0.586-0.779, *P*<0.001). There was evidence of low heterogeneity of treatment effect among the studies for mortality, and none of the studies had a particular impact on the summary result. The result was not influenced by age, sex, or comorbidities. We identified low risk of publication bias related to this outcome.

**Conclusion:**

This review found that TAR presents the best long-term results in patients who undergo CABG. Given that many patients are likely to benefit from TAR, its use should be encouraged.

**Table:** 

Abbreviations, acronyms & symbols				
ART	= Arterial Revascularization Trial		MI	= Myocardial infarction
BIMA	= Bilateral internal mammary artery		NA	= Not available
CABG	= Coronary artery bypass grafting		NM	= Non-multicenter
CENTRAL/CCTR	= Cochrane Central Register of Controlled Trials		NP	= Non-prospective
CI	= Confidence interval		NR	= Non-randomized
COPD	= Chronic obstructive pulmonary disease		P	= Prospective
EMBASE	= Excerpta Medica dataBASE		PRISMA	= Preferred Reporting Items for Systematic Reviews and Meta-analyses
HR	= Hazard ratio		PVD	= Peripheral venous disease
IMA	= Internal mammary artery		R	= Randomized
LILACS	= Literatura Latino-Americana em Ciências da Saúde		RA	= Radial artery
LIMA	= Left internal mammary artery		SciELO	= Scientific Electronic Library Online
LVEF	= Left ventricular ejection fraction		SIMA	= Single internal mammary artery
M	= Multicenter		SVG	= Saphenous vein graft
MEDLINE	= Medical Literature Analysis and Retrieval System Online		TAR	= Total arterial revascularization

## INTRODUCTION

### Rationale

Coronary artery bypass grafting (CABG) is one of the most commonly performed surgical operations worldwide and is currently considered as the revascularization strategy of choice for multivessel coronary artery disease^[1]^. However, optimal graft selection has been an important matter of debate. More specifically, graft failure considerably influences outcomes of CABG as it is associated with recurrent angina, poor survival, and need for reoperation^[2]^.

The superiority of the left internal mammary artery (LIMA) over the saphenous vein graft (SVG) to bypass a stenotic left anterior descending artery has long been established and is considered standard of care^[3]^. Furthermore, bilateral internal mammary artery (BIMA) grafts and/or radial artery (RA) grafts have been consistently shown to provide better results than SVG^[4,5]^. Three recently published meta-analyses have outlined the benefits of BIMA over single internal mammary artery (SIMA) in terms of long-term survival^[6-8]^.

Despite total arterial revascularization (TAR) being long advocated as the best revascularization strategy - although this is not a universal belief -, it remains underutilized. However, the recent Arterial Revascularization Trial (ART) demonstrated that, in the long-term, TAR has had the lowest rate of mortality and greatest reduction in complications when compared to single arterial graft or multiple arterial grafts^[9]^.

### Objectives

We set out to conduct a meta-analysis investigating long-term mortality (over 10 years) of TAR when compared to non-TAR. We followed the Preferred Reporting Items for Systematic Reviews and Meta-analyses (PRISMA) guidelines.

## METHODS

### Eligibility Criteria

With the population, intervention, comparison, outcomes, study design (or PICOS) strategy, studies were considered if: (1) the population comprised patients who underwent CABG; (2) there was a group of patients who underwent CABG with TAR (possible scenarios: "BIMA" or "BIMA+RA" or "SIMA+RA"); (3) there was a group of patients who underwent CABG with non-TAR (possible scenarios: "SIMA+SVG" or "BIMA+SVG" or "SIMA+RA+SVG" or "BIMA+RA+SVG); (4) outcomes included at least a 10-year follow-up; (5) studies were retrospective, prospective, randomized, or non-randomized; if non-randomized, the studies should be propensity-score matched studies.

### Search Strategy

Databases were searched for articles meeting our inclusion criteria and published by October 2020: Medical Literature Analysis and Retrieval System Online (or MEDLINE), Excerpta Medica dataBASE (or EMBASE), Cochrane Central Register of Controlled Trials (or CENTRAL/CCTR), ClinicalTrials.gov, Scientific Electronic Library Online (or SciELO), Literatura Latino-Americana em Ciências da Saúde (or LILACS), Google Scholar, and reference lists of relevant articles. We carried out the search with the following terms: "CABG OR Coronary Artery Bypass Grafting" AND "Total Arterial Grafting" OR "Total Arterial Revascularization" OR "arterial graft" OR "non-Total Arterial Revascularization" OR "non-total arterial grafting" OR "non-arterial graft".

The following steps were taken: (1) identification of titles of records through databases searching, (2) removal of duplicates, (3) screening and selection of abstracts, (4) assessment for eligibility through full-text articles, and (5) final inclusion in the study. Studies were selected by two independent reviewers. When concordance was absent, a third reviewer took the decision to include or exclude the study.

### Data Items

The primary endpoint was long-term mortality (at least a 10-year follow-up). Two independent reviewers extracted the data. When concordance was absent, a third reviewer checked them and took the final decision. From each study, we extracted patient characteristics, study design, and outcomes.

### Meta-Analysis

Pooled hazard ratio (HR) with 95% confidence interval (CI) and *P*-values for death were calculated. Forest plots were created to represent the primary outcome. Chi-square test and I^2^ test were performed for assessment of statistical heterogeneity^[10]^. The HR were combined across the studies using a weighted DerSimonian-Laird random effects model^[11]^. Funnel plots represent the analysis of publication bias, statistically analyzed by Begg and Mazumdar's test^[12]^ and Egger's test^[13]^.

### Sensitivity Analysis

The influence of a single study on the overall effect of TAR on the main outcome was assessed by sequentially removing one study (the "leave-one-out" method). This sensitivity analysis was carried out to test the consistency of these results in order to investigate if individual studies had an excessive impact on the results.

### Meta-Regression

Meta-regression analyses were performed to determine whether the effects of TAR were modulated by pre-specified factors. Meta-regression graphs describe the effect of off-pump CABG on the outcome (plotted as a log HR on the y-axis) as a function of a given factor (plotted as a mean or proportion of that factor on the x-axis). Meta-regression coefficients show the estimated increase in log HR per unit increase in the covariate. Since log HR > 0 corresponds to HR > 1 and log HR < 0 corresponds to HR < 1, a negative coefficient would indicate that as a given factor increases, the HR decreases.

The pre-determined modulating factors to be examined were age, male sex, hypertension, diabetes, hyperlipidemia, chronic obstructive coronary disease, peripheral vascular disease, left ventricular ejection fraction < 50%, and myocardial infarction.

A two-tailed *P*-value < 0.05 was considered statistically significant. All analyses were completed with R Statistical Software (version 3.6.3, Foundation for Statistical Computing, Vienna, Austria).

## RESULTS

### Study Selection

**Fig. 1:**
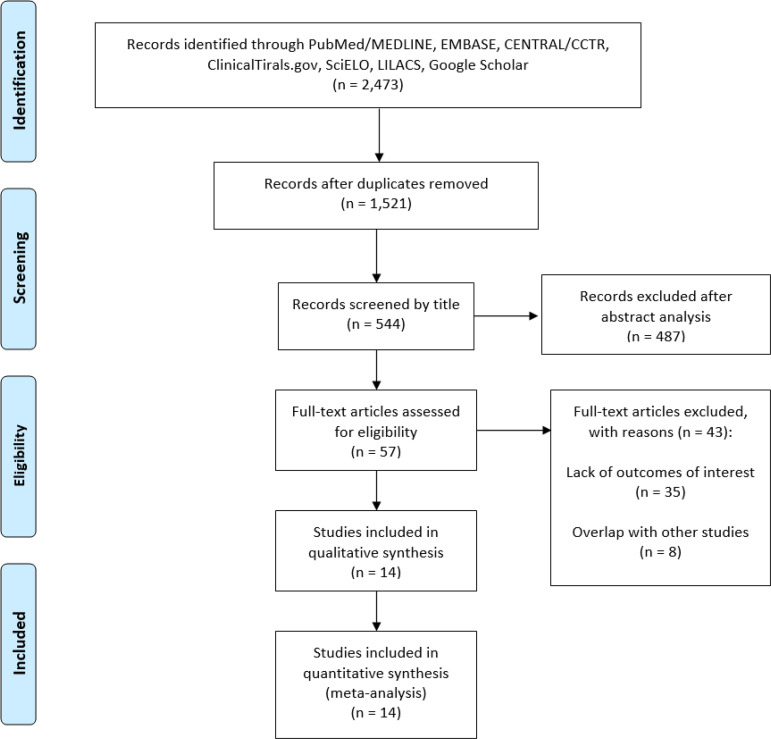
Flow diagram of studies included in data search. CENTRAL/CCTR=Cochrane Central Register of Controlled Trials; EMBASE=Excerpta Medica dataBASE; LILACS=Literatura Latino-Americana em Ciências da Saúde; MEDLINE=Medical Literature Analysis and Retrieval System Online; SciELO=Scientific Electronic Library Online.

A total of 2,473 citations were identified, of which 57 studies were potentially relevant and retrieved as full-text. Fourteen publications^[9,14-26]^ fulfilled our eligibility criteria. Interobserver reliability of study relevance was excellent (Kappa=0.86). The PRISMA Flow Chart is presented in Figure 1.

### Study Characteristics

Detailed characteristics of the studies and their populations are listed in Table 1. A total of 22,746 patients (TAR: 8,941 patients; non-TAR: 13,805 patients) were included from studies published from 2012 to 2020. The studies consisted of patients whose mean age was around 65 years and who were most often male. One study was a post-hoc analysis of a randomized controlled trial, one study was prospective, and four were multicenter. All the observational studies had propensity-score matched groups.

**Table 1:** Characteristics of the studies and populations.

		Number of patients		Age, years (mean)		Male sex (%)		Arterial hypertension (%)		Diabetes mellitus (%)		Dyslipidemia (%)		COPD (%)		PVD (%)		LVEF < 50% (%)		MI (%)	
Study	Study design	TAR	Non-TAR	TAR	Non-TAR	TAR	Non-TAR	TAR	Non-TAR	TAR	Non-TAR	TAR	Non-TAR	TAR	Non-TAR	TAR	Non-TAR	TAR	Non-TAR	TAR	Non-TAR
Taggart et al.^(9)^	P, R*, M	843	2025	64.0	64.0	85.6	87.2	78.5	78.1	23.8	23.1	93.2	93.7	1.3	2.8	6.7	6.9	26.8	24.6	43.4	43.7
Rocha et al.^(14)^	NP, NR, M	2132	2132	61.9	62.0	83.0	83.0	68.2	65.7	29.4	30.0	72.0	73.1	6.6	7.9	10.4	10.6	32.9	33.3	27.9	28.1
Obed et al.^(15)^	NP, NR, NM	81	81	66.1	66.3	70.0	72.0	NA	NA	21.0	23.0	58.0	56.0	8.0	6.0	NA	NA	NA	NA	NA	NA
Formica et al.^(16)^	NP, NR, NM	190	190	60.7	59.5	86.8	87.9	85.8	84.7	21.6	17.9	71.6	72.1	4.7	3.2	NA	NA	NA	NA	23.7	23.2
Grieshaber et al.^(17)^	NP, NR, NM	98	152	63.0	66.0	79.0	78.0	92.0	95.0	31.6	30.3	68.0	66.0	8.2	11.0	NA	NA	38.0	52.0	100	100
Royse et al.^(18)^	NP, NR, NM	232	232	67.0	67.7	78.0	78.0	60.0	61.0	17.0	16.0	66.0	70.0	12.0	12.0	12.0	11.0	NA	NA	50.0	51.0
Bisleri et al.^(19)^	NP, NR, NM	175	175	76.0	76.0	73.2	72.0	78.2	72.0	46.9	23.8	60.5	60.5	13.7	58.2	NA	NA	31.4	28.6	45.1	37.7
Bisleri et al.^(20)^	NP, NR, NM	151	151	74.0	76.0	76.0	83.0	35.0	38.0	25.0	27.0	41.0	34.0	18.0	14.0	NA	NA	NA	NA	31.0	29.8
Mohammadi et al.^(21)^	NP, NR, NM	249	249	56.1	55.8	90.4	88.8	53.8	52.8	12.1	13.7	92.0	91.6	4.4	6.8	10.4	11.2	NA	NA	47.8	48.6
Navia et al.^(22)^	NP, NR, NM	485	485	65.4	65.5	87.0	86.0	76.0	76.0	27.0	29.0	73.0	71.0	4.7	4.5	4.5	2.3	15.0	15.7	26.0	25.8
Shi et al.^(23)^	NP, NR, M	262	262	60.0	60.0	90.0	91.0	50.0	50.0	13.0	13.0	NA	NA	NA	NA	5.0	5.0	22.0	22.0	44.0	44.0
Suzuki et al.^(24)^	NP, NR, NM	250	260	69.0	70.8	82.6	78.5	72.3	65.4	54.0	41.2	56.3	45.4	19.8	21.2	NA	NA	NA	NA	31.5	43.5
Garatti et al.^(25)^	NP, NR, NM	209	243	48.8	50.0	95.0	96.0	36.0	43.0	15.0	14.0	67.0	65.0	4.0	3.0	23.0	11.0	NA	NA	69.0	69.0
Nasso et al.^(26)^	NP, NR, M	3584	7168	67.1	67.1	79.9	79.1	NA	NA	48.0	48.3	NA	NA	10.0	10.0	NA	NA	33.1	32.9	12.6	12.4

COPD=chronic obstructive pulmonary disease; LVEF=left ventricular ejection fraction; M=multicenter; MI=myocardial infarction; NA=not available; NM=non-multicenter; NP=nonprospective; NR=non-randomized; P=prospective; PVD=peripheral venous disease; R=randomized; TAR=total arterial revascularization

* Post-hoc analysis

### Synthesis of Results

**Fig. 2:**
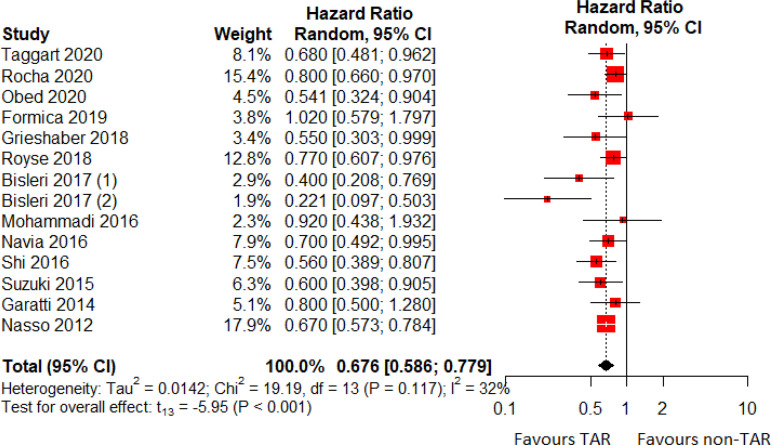
Forest plot for long-term mortality. CI=confidence interval; TAR=total arterial revascularization

**Fig. 3:**
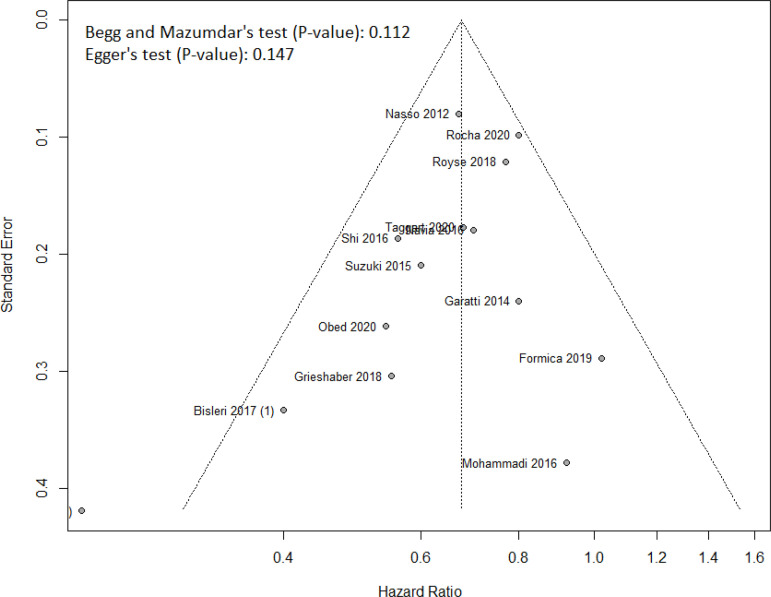
Funnel plot analysis of publication bias for long-term mortality

The HR for mortality in the TAR group compared with the non-TAR group for each study is reported in Figure 2. There was evidence of low heterogeneity (I^2^=32%, *P*=0.117) of treatment effect among the studies for mortality. We observed lower rates of mortality in the TAR group (random effect model: HR 0.676, 95% CI 0.586-0.779; *P*<0.001). Funnel plot analysis disclosed no asymmetry around the axis for the outcome, suggesting low risk of publication bias related to this outcome (Figure 3).

### Sensitivity Analysis

**Fig. 4:**
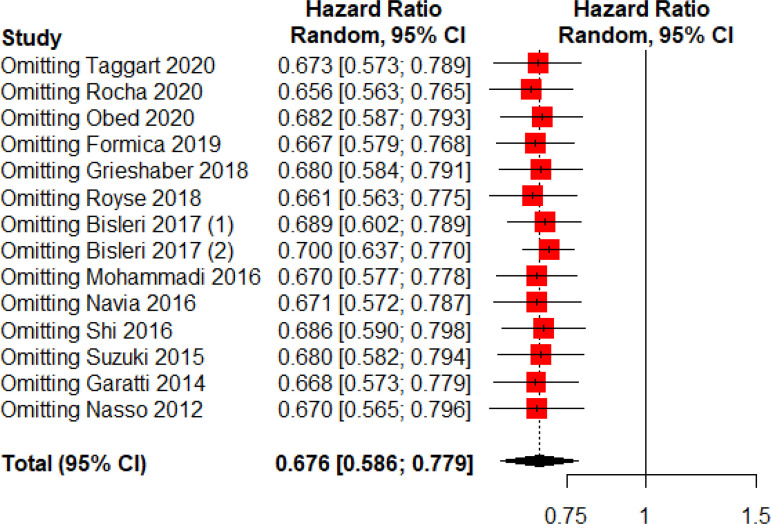
Sensitivity analysis (leave-one-out). CI=confidence interval

Sensitivity analyses performed by removing each single study from the meta-analysis (in order to determine the influence of individual data sets on the pooled HRs) showed that none of the studies had a particular impact on the summary results of mortality (Figure 4).

### Meta-Regression Analysis

None of the predetermined modulating factors showed any correlation with the studied outcomes, suggesting that the results were not influenced by any of these factors.

## DISCUSSION

### Summary of Evidence

To the best of our knowledge, this is the largest meta-analysis of randomized controlled trials and propensity-matched observational studies performed to date that investigated long-term mortality of TAR *versus* non-TAR. Our findings provide additional value by demonstrating that, after a follow-up of over 10 years, patients undergoing CABG surgery with TAR had lower risk of mortality compared to those undergoing non-TAR CABG.

### Comments

Surgical treatment of patients with multivessel coronary artery disease has made important progress in the last decades. Since it has become accepted that complete revascularization provides a benefit compared to incomplete revascularization^[2]^, several techniques have been developed. One option is to use SIMA and complete the revascularization using SVG. However, given the threefold higher risk of graft failure with venous compared to of arterial conduits^[27]^, this strategy might be hampered by recurrent angina, poor survival, and need for reoperation. Another option, which has increasingly gained attention, is to complete the revascularization using arterial conduits uniquely by means of BIMA and/or RA grafts. Nonetheless, TAR largely remains ignored. In the United States of America, multi-arterial CABG constitutes only 10% of CABG done using ≥ 2 arterial grafts, and < 1% with three arterial grafts^[28]^. Clearly, several challenges remain.

One of the reasons of concern about TAR is the use of the RA. It is prone to vasospasm, technically more challenging, and carries the risk of radial nerve injuries, refraining surgeons from adopting it as a conduit^[29,30]^. Yet, besides its superior patency rates over venous grafts^[31-33]^, several characteristics qualify RA as an adequate conduit for CABG: its resistance to atherosclerosis, accommodation to arterial pressure, possibility of parallel LIMA harvesting, caliber size and length, and the considerable muscular wall enabling easy handling^[34]^.

Another reason for reluctance is the perceived increased risk of deep sternal wound infection with BIMA harvesting. In this regard, internal mammary artery (IMA) skeletonization may help minimize sternal complications, as it causes minimal trauma on the chest wall in comparison to conventional pedicled IMA, leading to lower risk of sternal complications^[35,36]^. Furthermore, in terms of flow capacity, a skeletonized IMA appears to be superior in comparison with a pedicled IMA during CABG^[37]^. In terms of patency, skeletonized IMA appears to be non-inferior in comparison to pedicled IMA after CABG^[38]^. Of note, the wound complication rate with skeletonized BIMA was the same as that of a pedicled SIMA in the ART^[9]^.

As demonstrated in our meta-analysis, TAR had a significant survival benefit on a long-term follow-up of over 10 years after CABG. In the above, we discussed some of the barriers that are currently still hindering wide implementation of TAR into the cardiac surgical landscape. We also showed how these can be addressed, thereby optimizing the benefits of TAR while minimizing the risk for its potential complications. It has to be noted that there will remain scenarios were vein grafts will still be needed or even preferable above arterial grafts, such as in patients with expected short survival, variant anatomy, or as a bailout in case of arterial graft failure. However, these specific scenarios will only make up a minority of CABG cases. The 90% of patients currently receiving only a single graft in the United States of America is therefore largely out of proportion. Given the findings of our meta-analysis, several of these patients might benefit from TAR.

### Role of RA in TAR

The Radial Artery Patency and Clinical Outcomes (or RAPCO) trials^[39]^ found that the 10-year patency rate of the RA was significantly better than that of the free right internal thoracic artery, and higher than that of the saphenous vein, although this latter difference was not statistically significant. Obed et al.^[15]^ demonstrated that using the RA and the left IMA as T-graft is associated with a significant long-term survival benefit in patients undergoing CABG and it might be a promising alternative to conventional use of a SIMA supplemented by SVG. Furthermore, Carneiro et al.^[40]^ demonstrated that the site of proximal anastomosis of the RA (either onto the aorta or as Y composite grafts) does not interfere in mid- and long-term graft occlusion and patency rates.

### Risk of Bias and Limitations

Since studies with statistically significant results are more likely to be accepted for publication in medical journals in comparison with those with null or non-significant results, there is always the risk of publication bias. Nevertheless, the impact of TAR on the outcomes in our meta-analysis has low probability of being under the influence of publication bias according to the statistical analyses. Only one of the included studies was a randomized controlled trial, for which we tried to compensate with the inclusion of observational studies with matched populations.

## CONCLUSION

This systematic review with meta-analysis found that TAR presents the best long-term results in patients who undergo CABG surgery. Given that many patients are likely to benefit from TAR, our findings encourage the use of TAR in a larger group of patients than is currently being performed.

**Table:** 

Authors' roles & responsibilities	
SCR	Substantial contributions to the conception of the work; and the analysis of data for the work; drafting the work and revising it critically for important intellectual content; final approval of the version to be published
JVDE	Substantial contributions to the conception and design of the work; and the analysis of data for the work; drafting the work and revising it critically for important intellectual content; final approval of the version to be published
LRPC	Substantial contributions to the conception of the work; and the analysis of data for the work; drafting the work and revising it critically for important intellectual content; final approval of the version to be published
ACEN	Substantial contributions to the conception of the work; revising it critically for important intellectual content; final approval of the version to be published
AAR	Substantial contributions to the conception of the work; revising it critically for important intellectual content; final approval of the version to be published
AA	Substantial contributions to the conception of the work; revising it critically for important intellectual content; final approval of the version to be published
WBF	Substantial contributions to the conception of the work; revising it critically for important intellectual content; final approval of the version to be published
AR	Substantial contributions to the conception of the work; revising it critically for important intellectual content; final approval of the version to be published
KZ	Substantial contributions to the conception of the work; revising it critically for important intellectual content; final approval of the version to be published
AW	Substantial contributions to the conception of the work; revising it critically for important intellectual content; final approval of the version to be published
DCSF	Substantial contributions to the conception of the work; revising it critically for important intellectual content; final approval of the version to be published
MPBOS	Substantial contributions to the conception and design of the work; and the analysis of data for the work; drafting the work and revising it critically for important intellectual content; final approval of the version to be published
